# Supramolecular Hydrogel Based on pNIPAm Microgels Connected via Host–Guest Interactions

**DOI:** 10.3390/polym10060566

**Published:** 2018-05-23

**Authors:** Iurii Antoniuk, Daria Kaczmarek, Attila Kardos, Imre Varga, Catherine Amiel

**Affiliations:** 1 University Paris Est, ICMPE (UMR 7182), CNRS, UPEC, F-94320 Thiais, France; iurii.antoniuk@gmail.com; 2 Institute of Chemistry, Eötvös Loránd University, Pázmány s. 1/A, 1117 Budapest, Hungary; daria.kaczmarek@nanos3.eu (D.K.); kardosattila87@gmail.com (A.K.); 3 Department of Chemistry, University J. Selyeho, 945 01 Komárno, Slovakia

**Keywords:** host–guest polymer complex, microgel, hydrogel, *β*-cyclodextrin polymer, rheology, temperature-induced sol–gel transition

## Abstract

In this work, host–guest supramolecular hydrogels were prepared from poly(*N*-isopropylacrylamide) (pNIPAm) microgels utilizing electrostatic and host/guest self-assembly. First, pNIPAm microgels bearing a poly(acrylic acid) (pAAc) shell were coated with positively charged *β*-cyclodextrin polymers. Addition of adamantane-substituted dextrans (Dex-Ada) allowed us to establish interparticle connections through *β*-cyclodextrin-adamantane (*βCD*-Ada) inclusion complex formation, and thus to prepare hierarchical hydrogels. Under the conditions of hydrogel formation, close contact between the microgels was ensured. To the best of our knowledge, this is the first example of doubly crosslinked microgels prepared by noncovalent crosslinking via host–guest interactions. The prepared macrogels were studied with rheology, and fast mechanical response to temperature variation was found. Furthermore, the hydrogels exhibit fully reversible temperature-induced gel–sol transition at the physiological temperature range (37–41 °C), due to the synergetic effect between shrinking of the microgels and dissociation of *βCD*-Ada crosslinks at higher temperatures. This opens up attractive prospects of their potential use in biomedical applications.

## 1. Introduction

Hydrogels are crosslinked 3D networks composed of hydrophilic polymeric chains or low-molecular-mass gelators [1]. Due to their structural and functional resemblance to extracellular matrix, hydrogels are widely used in biomedical industry as scaffolds in regenerative medicine, and depos for sustained release of therapeutics [2,3,4]. Incorporation of particulate drug-delivery vehicles, and thermoresponsive microgels in particular, in hydrogel matrices might increase the pharmacological performance of both components [4]. The resulting composite or “plum puddinG″ hydrogels often show better mechanical properties, and the biocompatibility of microgels/nanoparticles is increased by integrating the latter in the biocompatible hydrogel network. The hydrogel matrix can act as a diffusion barrier aimed at diminishing the “burst release” of drug load that is an issue observed for nanoscale carriers [5]. At the same time, fixation of the nanoparticles in the matrix prevents their migration or washing away from the site of action in vivo. Finally, multiple types of microgels carrying different payloads can be entrapped in the same gel matrix and simultaneously delivered [6].

Richtering and Saunders distinguish three types of composite hydrogels containing microgels [7]: (i) microgel-filled hydrogels; (ii) microgel-reinforced hydrogels and (iii) doubly crosslinked microgels (DX microgels). All these types of hydrogels exhibit structural hierarchy with two or more distinct length scales between the adjacent crosslinks represented by the correlation lengths. In the first two cases, microgels are either mechanically entrapped or covalently linked to the bulk hydrogel matrix. In the case of DX microgels, however, the particles are directly interlinked and no additional hydrogel matrix is required.

Sivakumaran et al. have recently reported nanocomposite hydrogels composed of thermoresponsive pNIPAm-*co*-pAAc microgels either entrapped or covalently linked to inert carboxymethyl cellulose hydrogel matrix [8]. The hydrogels showed capacity to facilitate temperature-triggered long-term release of bupivacaine hydrochloride, a cationic drug. Interestingly, both increase of internal crosslink densities and covalent linking of microgels to the matrix led to lower overall rates of drug release, which was ascribed to reduced kinetics of microgel deswelling at higher temperatures (37 °C). Similar microgel-reinforced systems were reported, composed of hyaluronic acid–glycidyl methacrylate (HA–GMA) matrix and covalently linked ethylene-oxide (EO)-based microgels [9], and HA hydrogel network and covalently integrated surface-modified HA-microgels [10,11]. However, the mechanical properties of the hydrogels did not benefit from the presence of microgels in these cases, which is apparently related to the quite high distance between the embedded particles.

Temperature-induced change in the mechanical properties was found for polyacrylamide (PAAm)-based composite hydrogels noncovalently filled with pNIPAm microgels [12] and for pNIPAM-based composite hydrogels covalently filled with pNIPAm nanogels [13]. At high temperatures (*t* > volume phase-transition temperature—VPTT—of the microgels), the collapse of the filler microgel particles led to a change in their nature from a soft to a hard filler.

As mentioned above, DX microgels are formed via direct interaction between the microgel particles in a swollen state, provided that their surface groups and dangling polymer chains are close enough to establish a link [7]. DX microgels were first described in 2000 by Hu et al. [14]. Their system was composed of epichlorhydrin-crosslinked pNIPAm-*g*-pAAc microgels. In this case and in many later works, secondary crosslinking of the microgels was done covalently, by functionalizing them with vinyl groups and using free-radical chemistry [15,16]. Such DX microgels were used as injectable formulations to reinforce degenerated intervertebral disc tissue [16]. The microgel crosslinking was performed directly in vivo and the resulting hydrogels had remarkably high toughness (G′ = 0.7–1.3 × 10^5^ Pa). However, because of the permanent character of the secondary crosslinking, such DX microgels lack reversibility and self-healing capacity. The aim of the present work is to design reversible DX microgels using noncovalent crosslinking based on host–guest interactions.

The introduction of cyclodextrins (CDs) as structural units of both chemical and physical hydrogels proved to be beneficial given the ease and versatility of CD functionalization (via multiple primary and secondary hydroxyls), their high biocompatibility and the opportunity to exploit the cyclodextrin–hydrophobic guest inclusion chemistry [17]. Over the last decade, a large variety of complex supramolecular hydrogels containing CDs have been developed [3]. In some of the recent examples, *βCD*-Ada host–guest chemistry (HG) was used as a means to crosslink soft nanoscale objects into noncovalent 3D hydrogel networks. Indeed, adamantyl moieties are very often used as guests because of their strong affinity toward *βCD* hosts (schematic structures in Figure S1). For instance, Himmelein et al. used hydrophobically self-assembled cyclodextrin vesicles as multivalent 3D joints which were converted to a gel by addition of adamantane-modified hydroxyethylcellulose [18]. Due to its reversible nature, their system showed shear-thinning/self-healing properties and was injectable through a syringe. Recently, pNIPAM-based microgels have been surface modified with host and guest moieties, and it has been shown that the microgels were able to selectively interact with each other via a lock-and-key mechanism leading to the design of self-sorting colloidal systems [19,20].

Kardos et al. have recently developed [21] a novel approach to prepare core-shell pNIPAm microgels with unrestricted shell composition, for example, microgel particles with pNIPAm core and 100% AAc shell. The pure polyelectrolyte shell composition was aimed at facilitating the strong interaction and complex formation with various cationic agents and at making the pH-induced changes in phase behavior more pronounced.

**Figure 1:**
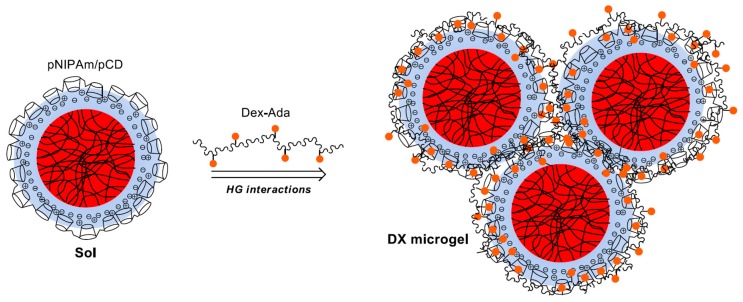
Schematic illustration of the strategy for host–guest-driven crosslinking of pCD-coated pNIPAm core (red)/AAc shell (blue) microgels into a 3D DX microgel network.

In this paper, we aimed at preparing microgel networks, which are crosslinked via host–guest interactions. Electrostatic interactions between pNIPAm–shell–pAAc microgels and positively charged *β*-cyclodextrin polymers (pCD) allowed the formation of pNIPAm/pCD core-shell microgel complexes. These *βCD*-coated microgels were subsequently used as a building block for hierarchical DX microgel formation by noncovalent crosslinking with adamantane-substituted dextrans (Dex-Ada). The Dex-Ada polymer was expected to establish interparticle connections between the microgels through the *βCD*-Ada inclusion complexation (Figure 1).

## 2. Experimental Section

### 2.1. Materials and Reagents

*N*-isopropylacrylamide (NIPAm), methylenbisacrylamide (BA), ammonium persulfate (APS), acrylic acid (AAc), 4-dimethylaminopyridine (DMAP), pyridine, 1-adamantanecarbonyl chloride, anhydrous grade *N*,*N*-dimethylformamide (DMF) and sodium dodecyl sulfate (SDS) were purchased from Sigma-Aldrich. *N*-isopropylacrylamide was recrystallized from hexane, methylenbisacrylamide was recrystallized from acetone and kept in a freezer usually for a few days before they were used for the synthesis of the microgel particle. To remove the inhibitor from the acrylic acid it was vacuum distilled and used immediately. Lithium chloride (Sigma-Aldrich, Lyon, France) and dextrans (*M_w_* 115 kDa (Dex_115_), 500 kDa (Dex_500_), Amersham, Umeå, Sweden) were dried overnight under vacuum at 80 °C. All other materials were used as received. All solutions were prepared in ultraclean Milli-Q water (total organic content = 4 ppb; resistivity = 18 mΩ·cm, filtered through a 0.2 µm membrane filter to remove particulate impurities).

### 2.2. Microgel Synthesis

Microgels composed of crosslinked poly(*N*-isopropylacrylamide) (pNIPAm) core and 100% poly(acrylic acid) (pAAc) shell were prepared using a semibatch precipitation polymerization technique developed by Kardos et al. [21]. In a typical polymerization reaction, a calculated amount of NIPAm was dissolved in Milli-Q water. Calculated volumes of a BA crosslinker stock solution and SDS stock solution were added to the NIPAm solution and the total volume of the mixture was adjusted to reach the desired final volume. The solution was introduced in a double-wall Pyrex glass reactor and it was stirred vigorously. To keep the temperature of the reaction mixture at constant 80 °C, the outer shell of the reactor was connected to a temperature bath and controlled temperature water was circulated in it. The reaction mixture was degassed by purging it with nitrogen for 60 min. Then, the reaction was initiated by adding a small volume of aqueous APS solution to the reactor. After reaching 95% conversion of the NIPAm monomer, the AAc monomer was fed into the reaction mixture (using a calculated volume of degassed aqueous AAc stock solution) to form the pure AAc-shell on the crosslinked pNIPAm microgel core. The final product, pNIPAm–shel–100%pAAc microgel, was purified from unreacted monomers and polymeric byproducts by ultracentrifugation (Beckman Optima XPN ultracentrifuge, 362,000 g), decantation, and redispersion. The centrifuged microgels were redispersed in Milli-Q water and the cycle was repeated up to 5 times. Finally, the purified microgels were freeze-dried and stored in a freezer before further use. The acrylic acid content of the prepared microgel particles was determined by conductometric titration in nitrogen atmosphere.

### 2.3. Cationic Poly(β-cyclodextrin) (pCD) Synthesis

pCD was prepared in a two-step procedure according to a previously described method [22,23]. Briefly, neutral epichlorohydrin-*βCD* polymers were first prepared by reacting *β*-cyclodextrin and epichlorohydrin in alkaline media. Then, these polymers were cationized by reaction with glycidyltrimethylammonium chloride. The success of the reaction was confirmed by ^1^H NMR (Bruker, Champs sur Marne, France, Avance II Ultrashield Plus 400 MHz NMR spectrometer). ^1^H NMR was also used to determine the *βCD* content and the number of cationic groups per cyclodextrin (N+/CD) in the prepared pCD polymer, using the approach previously described by our group [24]. The molecular weight (*M_w_*) was determined by size-exclusion chromatography. Size-exclusion chromatography coupled to multi-angle laser light scattering (SEC-MALLS) was performed in deionized water with 0.1 mol∙L^−1^ LiNO_3_ (0.05% NaN_3_) on TSK-gel type SW4000-3000 columns and detection by a Wyatt Dawn 8+ light-scattering detector and a Wyatt Optilab Rex refractive index detector.

### 2.4. Adamantane-Modified Guest Polymers

Adamantane grafted dextrans (Dex-Ada) were prepared via esterification reaction according to the procedure described elsewhere [25]. In a typical procedure, calculated amounts of dextrans with molecular weights of either Dex_115_ or Dex_500_ and lithium chloride were dissolved under stirring at 80 °C in anhydrous DMF. After the addition of calculated amounts of DMAP, pyridine and 1-adamantanecarbonyl chloride, the reaction mixture was left for 3 h at 80 °C and 15 h at room temperature. The polymers were isolated by precipitation into 2-propanol and filtration on sintered-glass funnel. The pure Dex-Ada samples were obtained by dialysis of the polymers’ concentrated water solutions against water, followed by freeze-drying. The degree of substitution by adamantyl groups in mole % per glucose unit was determined by ^1^H NMR in deuterated dimethylsulfoxide from the ratio of the integration of the protons of adamantyl groups and of the integration of anomeric and hydroxylic protons, as described in our earlier work [26].

### 2.5. Preparation of βCD-Coated Microgel Particles

(*pNIPAm*–*shell*–*pAAc/pCD core-shell microgel complexes*): In a typical procedure, 2 mL of pNIPAm–shell–pAAc microgel solution at 0.145 or 0.29 mM of negative charges (acrylic acid monomer concentration) were mixed with 2 mL of pCD solutions with calculated concentration of positive charges. The pCD solution was added to the stirred microgel solution by an automatic pipette and the mixture was stirred for an additional 2 min at 600 rpm at room temperature (pH = 7.0).

### 2.6. Supramolecular Hydrogel Preparation

Aqueous dispersions of pNIPAm–shell–pAAc/pCD (c_AAc_ = 6 mM, c(+)/c(−) = 0.8–2.0) coated microgels were mixed with aqueous solutions of Dex-Ada at pH 7 at room temperature. The obtained mixtures were then concentrated under vacuum at 55 °C (for further details see Discussion). Transition from turbid liquids to transparent gels occurred upon cooling the concentrated samples to 25 °C.

### 2.7. Methods and Instrumentation

#### 2.7.1. Rheology Measurements

Rheological characterization of the hydrogels was performed with a DHR-2 Rheometer (TA Instruments, Guyancourt, France), equipped with a 1° cone geometry of 40 mm diameter. An insulated ring was placed around the geometry to prevent water evaporation. The hydrogel sample under investigation (V = 400–500 μL) was placed on the center of the Peltier plate with a spatula or with a pipette (in the case of weak gels or viscous liquids) and the geometry was applied. Oscillatory frequency sweep measurements at 20 °C and 0.1% strain were performed in order to evaluate storage G′ and loss G″ moduli as a function of frequency in the range from 0.01 to 100 Hz. Limits of linear viscoelasticity regime were studied by strain amplitude sweep experiments with the strain amplitudes varying from 0.001% to 1000% (f = 10 Hz). Temperature sweep experiments from 25 to 62 °C were performed at a heating rate of 2 °C/min (f = 10 Hz, γ = 0.1%). In heating–cooling cycles experiment G′ and G″ were monitored as a function of time at two alternately changing temperatures, 25 and 50 °C (f = 10 Hz, γ = 0.1%).

#### 2.7.2. Dynamic Light Scattering (DLS)

Particle size and polydispersity were determined by DLS in pure water, microgel concentration of 10–20 ppm and temperature 25 °C. The measurements were performed with a Brookhaven Instruments (Holtsville, NY, USA) device, which consists of a BI-200SM goniometer and a BI-9000AT digital autocorrelator. A Coherent Genesis MX488-1000 STM laser was used as a light source. The laser was used at a wavelength of 488.0 nm and it emitted vertically polarized light. The autocorrelator was set in a “multi *τ*” mode; that is, the time axis was logarithmically spaced to span the required correlation time range. The autocorrelation functions were measured at a detection angle of 90° with a 100 μm pinhole size. The obtained autocorrelation functions were then analyzed by a second-order cumulant and the CONTIN methods. The extracted diffusion coefficients (D) were converted into hydrodynamic diameters $d_{h}$ using the Stokes–Einstein equation.

#### 2.7.3. Electrophoretic Mobility

The electrophoretic mobility measurements were performed using a Malvern Zetasizer NanoZ instrument. The data were analyzed using the M3-PALS technique. The standard error in the values of the electrophoretic mobility was around 10%, and measurements were always performed on freshly mixed samples.

#### 2.7.4. Cryo-Transmission Electron Microscopy (Cryo-TEM)

Cryo-TEM experiments were performed in Sorbonne University, Institut de Minéralogie, de Physique des Matériaux et de Cosmochimie, with a kind assistance by Jean-Michel Guigner. The images were recorded on an Ultrascan 2k CCD camera (Gatan, Pleasanton, CA, USA), using a LaB6 JEOL JEM 2100 (JEOL, Akishima, Tokyo, Japan) cryo-microscope operating at 200 kV with a JEOL low-dose system (Minimum Dose System, MDS) to protect the thin-ice film from any irradiation before imaging and to reduce the irradiation during the image capture. The images were recorded at 93 K. The samples were prepared as follows. A drop of the microgel complexes solution at pH 2 was deposited on a Quantifoil grid (Micro Tools GmbH, Jena, Germany). The grids were previously treated by glow-discharge in the presence of argon to increase their surface hydrophilicity. The excess of solution was then blotted out with a filter paper, and before evaporation the grid was quench-frozen in liquid ethane to form a thin vitreous ice film. The grid was mounted in a Gatan 626 cryo-holder cooled with liquid nitrogen and transferred in the microscope.

## 3. Results and Discussion

### 3.1. Preparation of Host-Molecule Functionalized Microgels

To facilitate the formation of 3D host/guest macrogels using pNIPAm microgels as the building blocks we prepared host-molecule-decorated microgels and guest-molecule-grafted polymers. We hypothesized that in the mixture of these materials, the host-molecule-decorated microgels can act as multivalent crosslinkers and enable the formation of a 3D gel matrix. However, it could be expected that efficient gel formation can take place only if the surfaces of the microgel beads are functionalized by the host molecules in high-enough density. To achieve high *βCD* coverage on the pNIPAm microgel surface, we used polyelectrolyte complex formation between pNIPAm–shell–AAc microgels and cationic *β*-cyclodextrin polymers (pCD, *M_w_* = 238 kDa, with 1.62 positive charge per *βCD*). The characteristics of these compounds are summarized in Table 1 where c_MG_, c_AAc_, c_CD_, c_N+_, c_Ada_ are the concentrations in microgel, AAC, CD, N+ and Ada respectively and n_N+_/n_CD_ is the molar ratio of N+ over CD. The main advantage of this approach is that the interaction of the highly charged non-crosslinked acrylic acid shell of the microgel and the small positively charged pCD coils leads to the accumulation of the pCD on the surface of the microgel and exposes the *βCD* moieties for host–guest interactions.

In order to utilize the pCD-covered microgels in host–guest gel formation, first we had to understand the phase behavior of the microgel/pCD mixtures. We found that upon mixing the microgels and pCD dilute solutions (60 ppm for the microgel) in stoichiometric ratio (1:1 ratio of negative charges of the pAAc monomers to the positive charges of the pCD), fast aggregation of the components took place resulting in a high-turbidity mixture. The turbidity of the samples decreased dramatically upon aging, as precipitate settled to the bottom of the container leaving a polymer-free transparent supernatant. Electrophoretic mobility measurements indicated that the fresh aggregates formed during mixing were charge–neutral, confirming the formation of colloidally unstable complexes.

**Figure 2:**
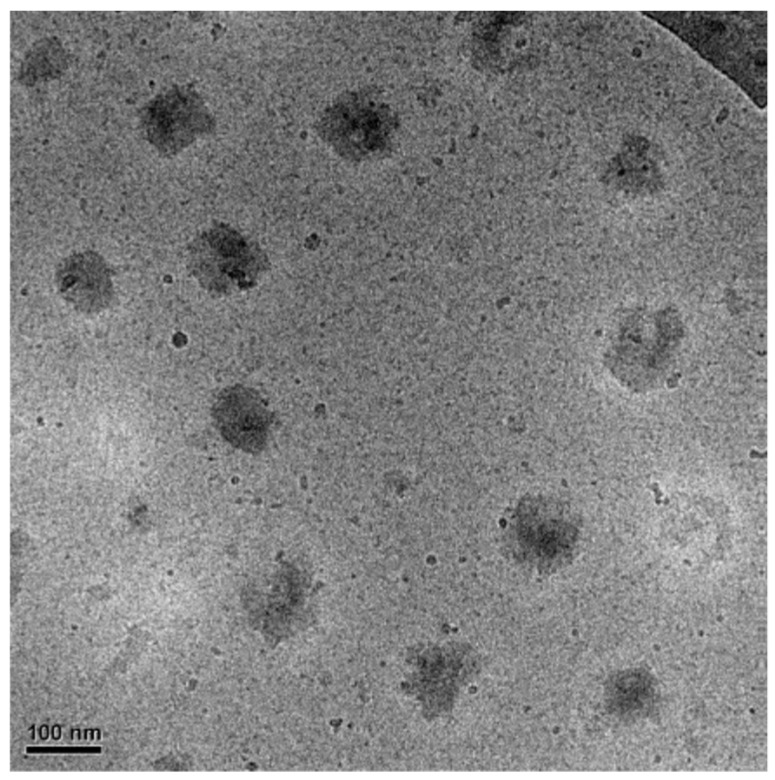
Cryo-transmission electron microscopy (cryo-TEM) image of microgel/pCD complexes prepared using pCD excess (c(+)/c(−) = 2). The scale bar indicates 100 nm.

However, when pCD was added to the microgel solution either in excess or in lower-than-stoichiometric positive-to-negative charge (c(+)/c(−)) ratios, the turbidity quickly decreased and transparent, stable mixtures formed. In the case of microgel excess, the c(+)/c(−) ratio had to be decreased to 0.8 or below to avoid precipitation. These samples had negative electrophoretic mobility indicating the excess of polyacrylic acid chains in the shell of the microgel/pCD complexes. In the case of pCD excess, the (c(+)/c(−)) ratio had to be increased to 1.5 to avoid precipitation. The microgel/pCD complexes had a positive electrophoretic mobility (~1 × 10^−8^ m^2^·V^−1^·s^−1^) showing that pCD is in excess of pAAc in the microgel shell. However, if the positive-to-negative ratio was further increased to 2, the electrophoretic mobility could be increased significantly further (~2 × 10^−8^ m^2^·V^−1^·s^−1^), demonstrating the formation of a more robust pCD shell on the microgel particles. To confirm that in excess pCD the formed microgel/pCD complexes are present as individual particles, cryo-transmission electron microscopy (cryo-TEM) experiments were performed. As shown in Figure 2, in the case of excess pCD (2:1 positive-to-negative charge ratio), no sign of aggregation can be detected, but well-separated individual particles are present in the sample.

### 3.2. Hydrogel Formation

Having established that stable microgel/pCD complexes can be prepared, we aimed at using these complexes for the preparation of 3D host–guest gels. We aimed at using the pCD-coated microgels as multivalent crosslinkers of adamantane-grafted dextran (Table 1). It can be expected that gels could form only if the adamantane-grafted dextran can bridge the gap between the neighboring microgels and strong (multiple) bonds can form between the microgel particles. To meet these requirements, the volume fraction of the microgel particles has to be high enough to ensure the close proximity of the microgels. According to Senff and Richtering, the rheological properties and phase behavior of pNIPAm microgel suspensions are strongly dependent on their effective volume fraction ($\phi_{ef}$) [27]. For instance, by measuring zero shear viscosity of microgels at different temperatures below lower critical solution temperature (LCST) as a function of $\phi_{ef}$, they observed a sharp increase in viscosity at $\phi_{ef}>$ 0.5 [28]. The phenomenon was ascribed to the microgel soft spheres being close to contact and interacting with each other at these concentrations. Hence, we decided to use microgel samples for the 3D gel formation with this effective volume fraction ($\phi_{ef}$ = 0.5).

Previously it was found by dynamic light-scattering measurements [29] that the molar weight of microgel particles (MG) prepared with similar collapsed size (*d_h_* = 150 nm determined by DLS at 40 °C) always exceeded the value of *M*_MG_ ~10^8^ g·mol^−1^. Using this molecular weight as a lower limit and the hydrodynamic diameter of the pCD-coated microgels measured in their swollen state (305 nm at 25 °C), we could calculate a lower limit for the microgel concentration in terms of dry weight per solution volume required to reach the desired effective volume fraction ($\phi_{ef}$ = 0.5). Using this simple estimation, we found that 6.0 g/L microgel concentration (15 mM for acrylic acid monomer concentration) should be sufficient in the final mixture to ensure the close proximity of the microgel beads required for gel formation.

To connect the neighboring microgel particles by multiple host–guest interactions, preferably large-molecular-weight Dex-Ada polymer has to be used with high-enough graft density of adamantane in high-enough concentration. To meet these requirements, we used dextran molecules with a molecular weight of 115 kDa (~700 glucose units) and 4.7% graft density (ca. 30 adamantane moieties on each polymer chain), and the concentration of the Dex-Ada polymer was chosen to ensure at least 1:1 adamantane:CD ratio in the samples.

Unfortunately, when we tried to prepare the 3D gel network by the direct mixing of the concentrated components at room temperature, the loss of colloid stability of the system resulted in precipitation. To circumvent this limitation, an indirect gelation approach was applied. In the first step, stable *β*-cyclodextrin polymer-coated microgel particles (MG/pCD) were prepared at lower concentrations (typically c_AAc_ = 6 mM, c(+) = 4.8–12 mM in the final mixture) at room temperature. These coated microgels were mixed with the Dex-Ada polymer solution at close to stoichiometric Ada/CD molar ratios. Then, the resulting homogenous mixtures were concentrated in vacuum at 50 °C to reach the desired 6.0 g/L microgel concentration. After cooling down the concentrated mixtures to room temperature, the pNIPAm core of the MG/pCD complexes could reswell, leading to the formation of final samples.

**Figure 3:**
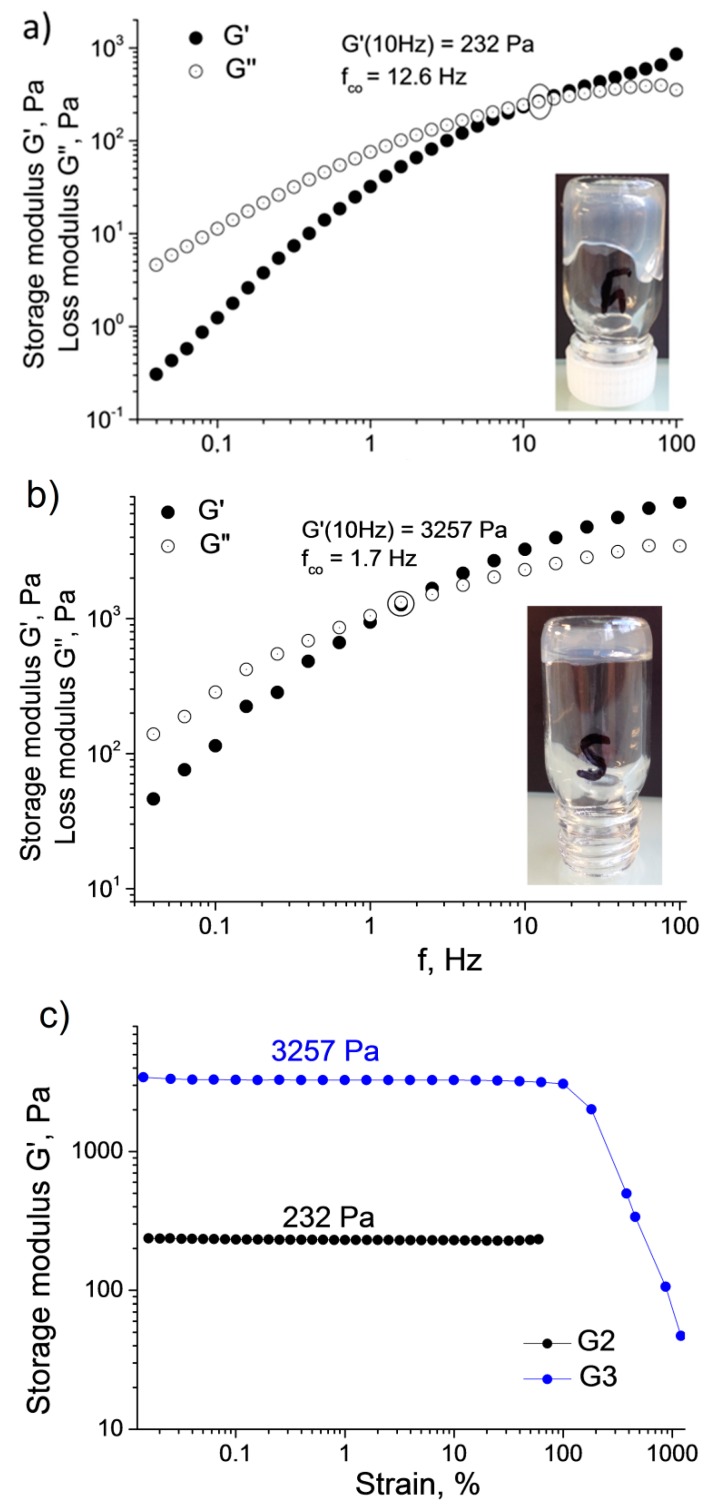
Oscillatory rheological measurements of hydrogel samples with varying pCD coverage of the microgel particles. Storage G′ and loss G″ moduli obtained from frequency sweep performed at 0.1% strain for (**a**) *G2*; (**b**) *G3*; (**c**) Storage modulus G′ of *G2* and *G3* samples obtained from amplitude-strain sweep performed at *f* = 10 Hz. All measurements were performed at 25 °C.

First, we investigated the effect of the pCD coverage of microgel particles on the gel formation. We used the three different surface coverages providing colloidally stable MG/pCD complexes: a low pCD coverage that provides stable complexes with acrylic acid excess in the shell (c(+)/c(−) = 0.8); a pCD coverage that provides stable complexes with a slight pCD excess in the shell (c(+)/c(−) = 1.5); and a full pCD excess where electrophoretic mobility reaches the plateau for the MG/pCD complexes (c(+)/c(−) = 2). In each case we used the same final microgel concentration (c_tot_ ~6 g/L), while the ratio of the CD and the adamantane moieties was kept at a fixed value (n_Ada_/n_CD_ ~ 1.3). For further details see Table 2. These experiments indicated that the pCD coverage of the microgel particles has a profound effect on the gel formation. In the case of the lowest pCD coverage (*G1*), precipitation occurred during the sample concentration. This can be explained by the loss of the colloid stability of the complex particles, whose charge could not counteract the increased van der Waals interaction among the collapsed complex particles (T > LCST). In the case of the overcharged complexes (excess pCD), no precipitation was observed and stable concentrated samples could be produced. At the same time, the lower pCD coverage (*G2*: c(+)/c(−) = 1.5) resulted in a viscous liquid sample, while the sample with the largest pCD coverage (*G3*) behaved as a physical gel (see insets in Figure 3). This indicates that although the MG/pCD complexes preserved their colloid stability in both cases during sample concentration, connectivity among the microgel particles could reach a percolation threshold only in the latter case due to the larger number of links performed by the adamantyl units, increasing the amount of Dex-Ada polymer being needed to ensure the constant adamantane/cyclodextrin ratio in the samples.

To confirm that the gel formation is indeed related to the host–guest complex formation, we prepared two control samples without the addition of the guest polymer (Table 2). Since it has been reported in the literature that uniform pNIPAm microgel dispersions with concentrations corresponding to close-packing (~5 wt %) undergo sol–gel transition below the LCST [30], we also concentrated the uncoated pNIPAm–shell–pAAc microgels to test if they can give a sol/gel transition in the investigated concentration range. This experiment indicated that the core/shell microgel particles did not form a gel even at concentrations as high as 10.1 wt % (*R1*), where the acrylic acid shells of the microgel particles strongly interpenetrate each other (semidilute systems). This can be explained by the strong electrostatic repulsion of the highly charged acrylic acid chains in the shell of the microgel particles (pH = 7).

In addition, the bare MG/pCD complexes (c(+)/c(−) = 2) were also concentrated to 10.5 wt % (*R2*), which resulted in the precipitation of the samples. The observed precipitation is a consequence of the loss of the colloid stability of the collapsed MG/pCD complexes. This clearly indicates that the colloid stability of the microgel complexes observed during the concentration of *G2* and *G3* samples is provided by the adamantane-grafted dextran chains and not exclusively by the charge of the MG/pCD complexes. Thus we can conclude that the host–guest complex formation indeed took place, and guest-polymer-wrapped hierarchical complexes formed upon mixing.

To get a better insight into the mechanical characteristics of the prepared samples, the viscoelastic properties of *G2* and *G3* were studied by oscillatory rheological measurements. Frequency sweep measurements of storage G′ and loss G″ moduli are shown in the two upper panels of Figure 3. The G′ and G″ values prove to be frequency dependent in both cases, which was previously reported as a typical behavior for associating systems [31,32,33]. At higher frequencies and shorter observation times, materials show gel character with the elastic component G′ dominating the viscous component G″, while at lower frequencies the system starts to behave as a viscous liquid with G″ > G′. In a number of earlier studies on host–guest polymeric hydrogels, such behavior is explained by the weak and reversible character of the host–guest links, which are being broken and reformed under applied stress [32,34,35].

The finite lifetimes of the interpolymer crosslinks are due to the fluctuations in the relative kinetic energy of the interacting adamantane and *βCD*-groups. These lifetimes ($\tau_{co}$), also known as relaxation times, may be estimated from the frequency (*f_co_*) of the crossover point of G′ and G″ in the frequency sweeps:

(1) $$\tau_{co}={\left(2\pi \cdot f_{co}\right)}^{-1}.$$

*f_co_* should decrease with the number of host–guest links between individual microgels. Indeed, the *G2* sample, which behaves as a viscous liquid (Figure 2a), has the higher $f_{co}$ value of 12.6 Hz ($\tau_{co}$ = 0.013 s), whereas in the case of *G3*, which behaves as a gel, the $f_{co}$ is shifted down to 1.7 Hz ($\tau_{co}$ = 0.094 s) (Figure 2b). The strength of the hydrogel structures was also studied by strain-amplitude sweep performed at f = 10 Hz (Figure 2c). The plateau G′ moduli vary from 232 Pa for *G2*, behaving as a viscous liquid, to 3257 Pa for the *G3* hydrogel. *G2* and *G3* exhibit quite high strain resistance and maintain their structure up to *γ* ~100% (Figure 2c). Similar values of elastic storage modulus were found for recently described cyclodextrin-based hierarchical hydrogels such as *βCD*-coated quantum dots crosslinked with azobenzene-modified thermoresponsive copolymers (G′ = 200–400 Pa, c_tot_ = 12.5 wt % and T > 40 °C) [36] or *βCD* vesicles interconnected with Ada-modified cellulose polymer (G′ = 200–400 Pa, c_tot_ = 2–3 wt %) [18].

We also attempted to fit the frequency sweep data of the samples by the Maxwell model. This basic viscoelasticity model was widely used for describing the behavior of entangled physically bonded polymer networks [37,38] and polymeric host–guest hydrogels [35]. The Maxwell model implies that storage G and loss G″ moduli of a viscoelastic body vary as a function of the strain angular frequency ω according to the following equations:

(2) $$G^{\prime }\left(\omega \right)=\frac{G_{0}{\left(\omega \tau \right)}^{2}}{1+{\left(\omega \tau \right)}^{2}},$$

(3) $$G^{\prime \prime }\left(\omega \right)=\frac{G_{0}\omega \tau }{1+{\left(\omega \tau \right)}^{2}},$$

where ω is the angular frequency [rad·s^−1^], τ is the relaxation time and $G_{0}$ is the limiting value of G′ at high frequencies where it typically reaches a plateau. From the equations above, it also follows that $G^{\prime }/G^{\prime \prime }=\;\omega \tau$. In our case, τ was obtained from the G′ and G″ crossover frequency value and was set as a fixed parameter during the fitting. Since G′ did not reach the plateau in the available range of angular frequencies, $G_{0}$ was set as a free optimized parameter. Despite the viscoelastic character of the samples evidenced by the frequency sweep data (Figure 3), rather poor fits to the Maxwell model were obtained. An example of such a fit for the *G3* sample is illustrated in Figure S3. Only the low-frequency part (0.1–1.6 rad·s^−1^) of the loss modulus G″ curve showed a reasonable agreement with the experimental data. The failure of the fitting might be related to the oversimplifications of the Maxwell model, which is suitable only for the description of the viscoelastic materials with a single and well-defined relaxation time, τ. For instance, van de Manakker et al. obtained relatively good fits for host–guest hydrogel networks composed of linear and four-arm star PEG polymers end-functionalized with *βCD* and cholesterol groups [35]. However, they found that the fitting to the Maxwell model was no longer possible for structurally more complex hydrogels composed of eight-arm PEGs with the same end-functionalities. They ascribed this discrepancy to the presence of a broader range of relaxation mechanisms with different relaxation times involved in the stress relaxation in the latter case. We assume that the same arguments hold for the structurally rather complex pNIPAm/pCD/DexAda doubly crosslinked microgels. Indeed, a good fit of the frequency sweep data has been obtained using a generalized Maxwell model using a distribution of relaxation times (Figure S3).

### 3.3. Influence of Host and Guest Polymer Characteristics on the Gel Formation

To develop a better understanding of the key parameters controlling the supramolecular gel formation, we also tested the effect of the host polymer (pCD) charge density on the gel formation. We used a host polymer to prepare the MG/pCD complexes, which had twice-as-large charge density (pCD3.2N+) as the host polymer used in the previous experiments (pCD1.6N+). This meant that while we could prepare the MG/pCD complexes with the same overall charge and colloid stability, the number of the CD groups present in the shell could be halved. The prepared complexes indeed preserved their colloid stability, however, when the system was concentrated in the presence of stoichiometric amount of guest polymer (n_Ada_/n_CD_ = 1), gel formation could not be observed. To facilitate the gel formation, we repeated the same experiment with doubling the MG concentration (*G4* in Table 2), thus ensuring that the same amount of adamantane moieties were present in the sample as in the case of the successfully gelled *G3* sample, while the increased microgel concentration resulted in tighter contact of the microgel particles. Surprisingly, the mixture remained a viscous liquid even at as high a total polymer content as c_tot_ = 14.8 wt %. Taking into account that doubling the charge density of the pCD polymer while keeping the charge ratio constant (c(+)/c(−) = 2) decreased the surface concentration of the pCD coverage by half, we hypothesized that the lack of gel formation was related to the decreased number of host/guest interactions of the guest polymers bridging the microgel particles.

In an attempt to overcome this problem, we used a guest polymer with increased number of grafted adamantane groups. This was achieved by using a larger-molecular-weight and graft density guest polymer (see Dex_500_Ada_6_, in Table 1) as a crosslinker. This polymer had ~200 grafted adamantane groups compared to the 32 groups of the previously used polymer (Dex_110_Ada_5_). This approach proved successful: A stable transparent gel was formed at c_tot_ = 11.0 wt % (*G5* in Table 2), confirming that the number of host–guest interactions established by the bridging polymer chains by the connected microgel particles is a key parameter in gel formation.

We also made frequency sweep rheological measurements on the prepared gel sample (*G5*). The measured curves (Figure S4) had the same qualitative features as measured previously for the *G3* sample (Figure 3), but we found a lower crossover frequency ($f_{co}$ = 1.1 Hz compared to the 1.7 Hz observed for *G3*), which represents a slower relaxation time ($\tau_{co}$ = 0.145 s vs. $\tau_{co}$ = 0.094 s for *G3*). This observation is in good agreement with the expected increased cooperativity of the interactions.

### 3.4. Thermoresponsive Behavior of the Supramolecular Gels

Since the obtained supramolecular hydrogels are composed of crosslinked thermoresponsive pNIPAm beads, their swelling and mechanical properties are expected to be temperature dependent. Previously, it has been found that hydrogels filled either noncovalently or covalently with pNIPAm microgels [12,13] became mechanically more robust at high temperatures (*t* > volume phase-transition temperature) due to changing the nature of the microgel particles from a soft to a hard filler. It has also been shown that the nanostructured gels formed by chemically crosslinked pNIPAm microgels or by metal–ligand interactions [39] shrunk with increasing temperature, and their volume deswelling closely mimicked the volume deswelling of the microgel building blocks.

**Figure 4:**
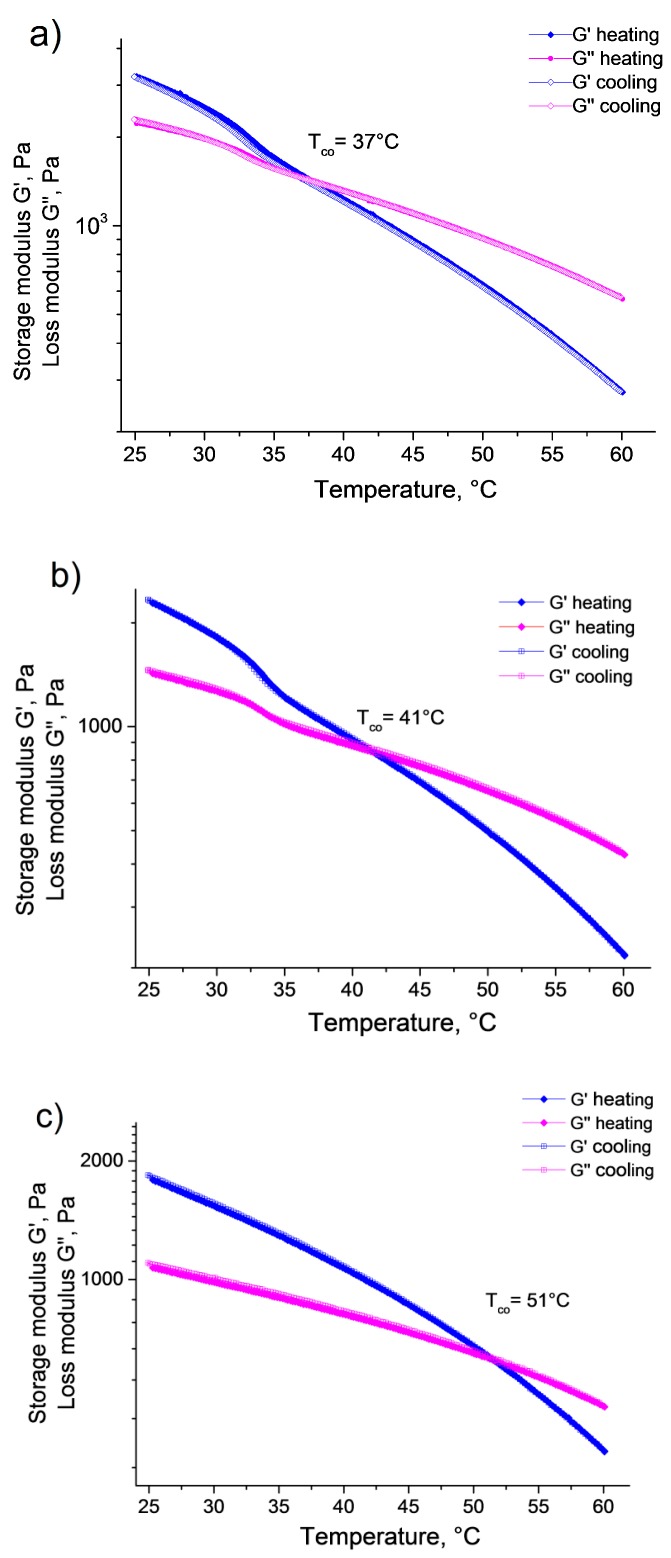
Evolution of storage G′ and loss G″ moduli of supramolecular gels under heating and cooling temperature ramps (temperature change rate = 2 °C/min, f = 10 Hz, *γ* = 0.1%) for (**a**) *G3*; (**b**) *G5*; (**c**) *R3* hydrogel samples.

Interestingly, the supramolecular gel samples we prepared did not shrink as a uniform body on temperature increase. Instead they remained a single-phase system with practically constant volume, and the gel sample turned into a liquid system at high temperatures. In order to follow the gel–sol transition of the supramolecular hydrogels, the storage and loss moduli were recorded as a function of temperature between 25 and 60 °C for both *G3* and *G5* (Figure 4). The measurements provided very similar results for the two gel samples. Both G′ and G″ decreased monotonously with increasing temperature. Furthermore, in the temperature range where the volume phase transition of the microgel particles occurs (~30 °C < *t* < 35 °C), the decrease of the storage and loss moduli accelerated then leveled off again, giving rise to a step-like decrease of the moduli. However, it is interesting to note this step-like decrease was significantly larger for G′ than for G″, decreasing the gap between the two curves. Since G′ decreased faster than G″, at some point G″ became larger (crossover temperature—*T_co_*), and the gel-to-sol phase transition occurred. It should also be noted that in the case of the *G3* sample, the crossover temperature was in a good agreement with the end of the temperature range where the pNIPAm/pCD microgels collapsed (VPTT: 37 °C), however, in the case of the *G5* sample, the crossover temperature was shifted to a higher value (41 °C) and it seemed independent of the microgel collapse. Finally, when the samples were gradually cooled back from 60 to 25 °C, the moduli showed full reversibility; hysteresis could not be observed neither for G′ nor for G″.

Contrary to previous investigations [12,13,39], our measurements clearly showed that the prepared supramolecular gels became softer with increasing temperature, and finally turned into a liquid at the crossover temperature. Since the main difference between the previously investigated samples and our supramolecular gels was that we used host–guest interaction for the crosslinking of the microgel particles, we assumed that the observed temperature dependence was related to the characteristics of the host–guest complex formation. Indeed, the group of B. Ravoo has also reported decreasing mechanical strength in the case of host–guest supramolecular hydrogels connected by vesicles [18].

To confirm this interpretation, we prepared a reference sample that had the same concentrations of the host and guest polymers as *G5* but did not contain microgel particles (*R3*). The storage and loss moduli recorded for this sample as a function of temperature are plotted in panel (c) of Figure 4. Similarly to the microgel-containing samples, G′ and G″ decreased monotonously with increasing temperature. However, the step-like steep decrease of the moduli in the temperature range of the microgel collapse was missing in this case, and as a consequence, the crossover temperature shifted to a much higher value (*T_co_* = 51 °C).

Based on these experimental results, it can be concluded that two independent processes take place in the supramolecular gel samples on temperature increase. On the one hand, as the volume phase-transition temperature is approached, the microgel building blocks shrink and become fully collapsed when the VPTT is reached. This should lead to the uniform shrinking of the gel samples and give rise to mechanically more-robust gels. However, this is counteracted by the fact that the host–guest association is an equilibrium process that has a negative enthalpy change (H). Thus, as the temperature is increased, the host–guest complex formation is shifted towards dissociation. This decreases the number of host–guest complexes in the system, and as a consequence, the average strength of the bonds bridging the microgel particles (the average number of host–guest complexes formed by a guest polymer) also decreases. This is evidenced by the continuously decreasing moduli and the gel–sol transition taking place even in the pure mixture of the host and guest polymers. At the same time, in the presence of the microgels, an osmotic effect attributed to the Dex-Ada chains opposes the deswelling of the gel sample. When the microgels shrink, the interstitial volume between the microgels is increased and the Dex-Ada chains accommodate this space to oppose the deswelling. This is the reason why there is no phase separation for these gels, contrary to the covalently crosslinked DX hydrogels [12,13,39]. As a consequence, the local Dex-Ada concentration decreases, inducing a decrease of the number of host–guest complexes. In a previous study of host–guest hydrogels, it has been shown that moduli were decreasing with the concentrations of host and/or guest moieties [29]. As a result, the average number of host/guest complexes formed by a guest polymer connecting the neighboring microgel beads decreases, and the supramolecular gel becomes softer, giving rise to the step-like decrease of moduli as the VPTT is approached.

The gel-to-sol transition temperature (*T*_CO_) corresponds to a temperature at which a percolation threshold in the connected microgel beads is attained. Increasing the number of adamantyl groups on the guest polymer should result in a larger number of host–guest complexes at any temperature, and thus to a shift of *T*_CO_ to larger temperature. This explains why the *T*_CO_ of *G3*, made with Dex_110_-Ada_5_ (32 adamantyl groups per chain), is lower by 4 °C than *T*_CO_ of *G5* made with Dex_500_-Ada_6_ (200 adamantyl groups per chain). An important message of this conclusion is that the gel–sol transition could be tuned within a wide temperature range by varying the size and graft density of the guest molecules, as well as by tuning the VPTT of the microgel beads.

**Figure 5:**
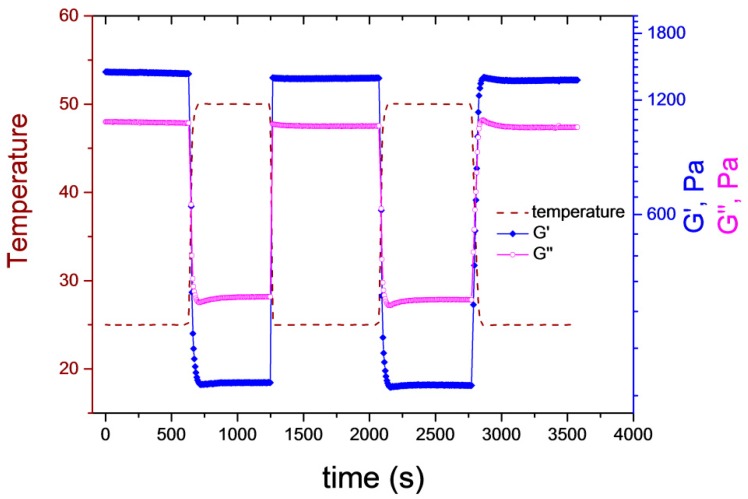
Oscillatory rheological measurements of *G3* under temperature ramps (*f* = 10 Hz, *γ* = 0.1%).

To test the response dynamics of the supramolecular gels, the sample was subjected to several heating–cooling cycles, where its temperature was alternated between 25 and 50 °C (Figure 5). As indicated by the figure, the temperature jumps required only a few seconds. G′ and G″ were recorded at a constant frequency (10 Hz). Both moduli changed promptly when the temperature jump was triggered, significant delay in their response to the temperature change could not be observed on the applied timescale. G″ was larger than G′ at 50 °C, whereas the inverse situation was obtained at 25 °C, meaning that a gel-to-sol transition occurred. The cycle was repeated several times, and the initial G′ and G″ values were fully restored in each case, proving the reversibility of the temperature-induced phase transition. One should note that the sample in Figure 5 has the same nominal concentration as G3, but the final concentrated sample was a softer gel at the end due to the uncertainties of the sample preparation. We are working on a more robust and simple sample-preparation method.

The observed fast response of the supramolecular hydrogel to the temperature change is in contrast with the observations made for the chemically crosslinked pNIPAM hydrogels, which have characteristic relaxation times on the order of 100 s under the same geometry conditions [40]. This can be explained by the fact that in this case, the overall volume of the gel does not change, thus the transport of the liquid out of and into the macroscopic gel structure is not required. Instead, the liquid transport is localized to the close neighborhood of the microgel beads and confined to a few-hundred-nanometer length scale. This renders the response time of the system to the subsecond timescale.

## 4. Conclusions

In summary, novel host–guest supramolecular hydrogels were prepared, containing pNIPAm–shell–pAAc microgels as responsive building blocks. We were able to define the conditions of supramolecular hydrogel formation in terms of the concentrations of the three components: The close contact between the microgel particles has to be ensured ($\phi_{ef}$ ≥ 0.5), pCD concentrations to give rise to a compact pCD shell formation is required (corresponding to twice the pCD concentration needed for stoichiometric MG/pCD complex formation), and close-to-stoichiometric *βCD*/Ada ratio is needed. To the best of our knowledge, this is the first example of DX microgels noncovalently crosslinked via host–guest interactions.

The material properties were studied with rheology and showed fast mechanical response to temperature variation. An important feature of these materials is their low deswelling sensitivity to temperature increase, contrary to most pNIPAM-based materials. This is due to the osmotic effect exerted by the Dex-Ada chains. Furthermore, the hydrogels exhibit fully reversible temperature-induced gel–sol transition due to the synergetic effect between shrinking of the microgels and dissociation of *βCD*-Ada crosslinks at higher temperatures. Due to the presence of microgels, the gel–sol transition temperature of pNIPAm/pCD/Dex-Ada is significantly shifted down to the physiological temperature range (37–41 °C) as compared to uniform p*βCD*N/DT-Ada host–guest hydrogels (51 °C). It opens up attractive prospects of their potential use in biomedical applications.

## Figures and Tables

**Table 1:** Characteristics of the synthetized polymers and microgels.

**Microgel (MG)**	**c_AAc_ in c_MG_**	**d_h_ (nm) (pH 7, 25 °C)**		
pNIPAm-shell-100%pAAc	1 mM in 0.40 g·L^−1^	450		
**Host polymers**	**c_N+_ in c_pCD_**	***M_w_* (kDa)**	**c_CD_ (wt %)**	**n_N+_/n_CD_**
pCD(1.6N+)	1 mM in 1.20 g·L^−1^	238	58.2	1.62
pCD(3.2N+)	1 mM in 0.68 g·L^−1^	268	51.8	3.24
**Guest polymers**	**c_Ada_ in c_DexAda_**	***M_w_* (kDa)**	**c_Ada_ (mol %)**	
Dex_115_-Ada_5_	1 mM in 3.68 g·L^−1^	115	4.7	
Dex_500_-Ada_6_	1 mM in 2.66 g·L^−1^	533	6.5	

**Table 2:** Molar ratios and weight concentrations of the components in supramolecular gel samples prepared from microgels (MG), pCD host polymers and Dex-Ada guest polymers.

Sample Code	Components	c(+)/c(−)	n_Ada_/n_CD_	c_MG_ (wt %)	c_pCD_ (wt %)	c_Dex-Ada_ (wt %)	c_tot_ (wt %)	Observation
*G1*	MG/pCD(1.6N+)/Dex_110_Ada_5_	0.8	1.25	0.6	1.4	3.4	5.4	phase separation
*G2*	MG/pCD(1.6N+)/Dex_110_Ada_5_	1.5	1.25	0.6	2.6	6.3	9.4	viscous liquid
*G3*	MG/pCD(1.6N+)/Dex_110_Ada_5_	2.0	1.25	0.5	3.3	8.1	11.9	gel
*R1*	MG	-	-	10.1	0	0	10.1	liquid
*R2*	MG/pCD(1.6N+)	2.0	-	1.5	9.0	0	10.5	phase separation
*G4*	MG/pCD(3.2N+)/Dex_110_Ada_5_	2.0	1.0	1.5	5.0	8.4	14.9	liquid
*G5*	MG/pCD(3.2N+)/Dex_500_Ada_6_	2.0	1.0	1.3	4.4	5.3	11.0	gel
*R3*	pCD(3.2N+)/Dex_500_Ada_6_	-	1.0	0	4.3	5.2	9.5	gel

## Supplementary Materials

The following are available online at http://www.mdpi.com/2073-4360/10/6/566/s1, Figure S1: Schematic structure of *β*-cyclodextrin and adamantyl moieties. Figure S2: Hydrodynamic diameter as a function of temperature for positively overcharged microgels. Figure S3: Frequency sweep data of *G3* fitted with a generalized Maxwell model. Figure S4: Oscillatory rheological measurements of *G5* sample.
