# Supplementary material for: Supramolecular Hydrogel Based on pNIPAm Microgels Connected via Host–Guest Interactions
Source: Polymers (Basel). 2018 May 23;10(6):566. doi: 10.3390/polym10060566 (PMC6403914; doi:10.3390/polym10060566)
Supplement: Supplementary file 1 [file polymers-10-00566-s001.pdf]

polymers

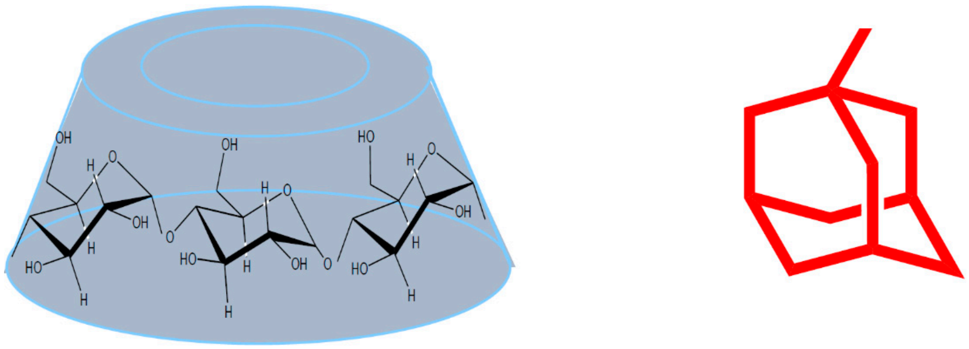

Figure S1. Schematic structure of β-cyclodextrin and adamantyl moieties.

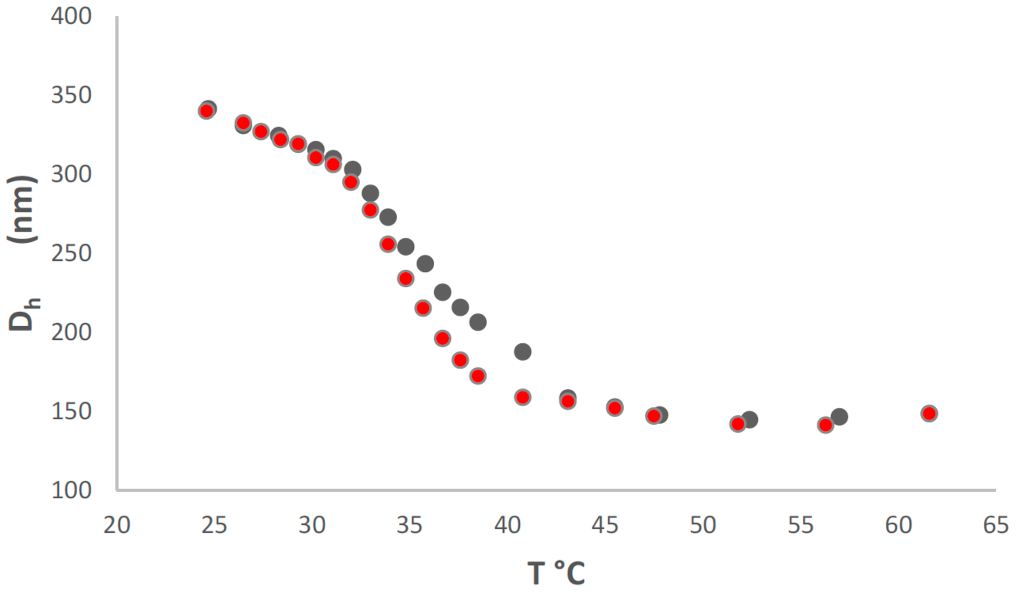

Figure S2. Hydrodynamic diameter as a function of temperature for positively overcharged microgels. MG/pCD(1.62N+) (c(+)/c(-)=2.2) at pH7 and C(-)=0.145 mM.

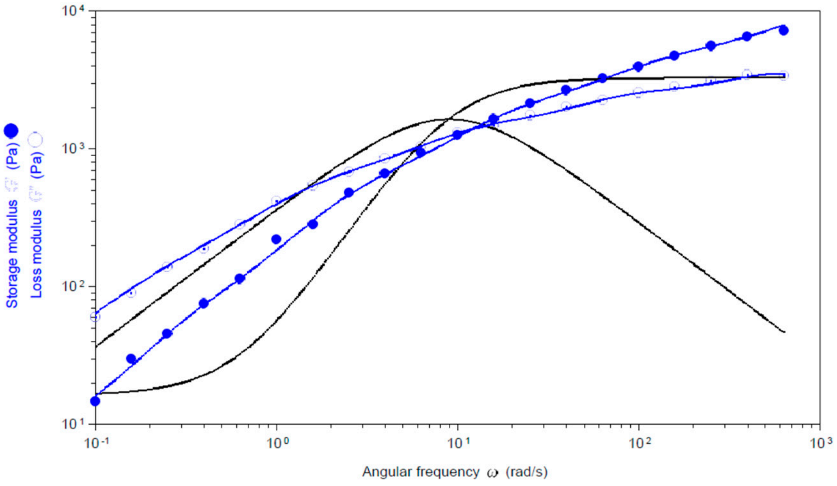

(**a**)

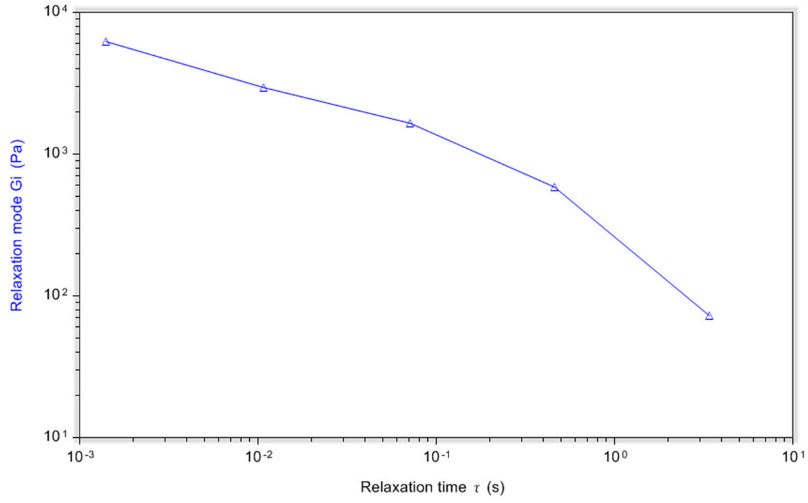

(**b**)

Figure S3. (**a**) Frequency sweep data (•,o) of sample G3 were fiited with a simple Maxwell model (black line) and with a generalized Maxwell model using a distribution of 5 correlation times (blue lines). (**b**) parameters of the generalized Maxwell model fit (G_0, τ) expressing a relaxation time distribution. (25°C, γ = 0.1%).

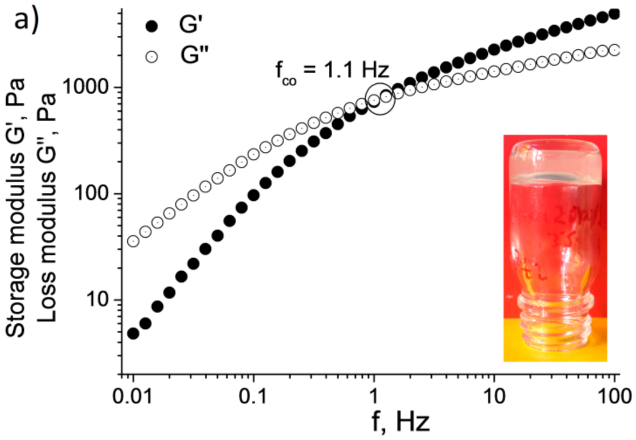

Figure S4. Oscillatory rheological measurements of hydrogels prepared using p*β*CD3.2N+ host and DT500Ada6 guest polymers. Storage G' and loss G'' moduli obtained from frequency sweep performed at 0.1% strain for G5. All measurements were performed at 25 °C.
